# Pharmacokinetics of ethambutol and weight banded dosing in South African adults newly diagnosed with tuberculosis and HIV

**DOI:** 10.1128/aac.01200-24

**Published:** 2024-12-23

**Authors:** Bonginkosi Ndzamba, Paolo Denti, Helen McIlleron, Peter Smith, Thuli Mthiyane, Roxana Rustomjee, Philip Onyebujoh, Juan Eduardo Reséndiz-Galván

**Affiliations:** 1 Division of Clinical Pharmacology, Department of Medicine, University of Cape Town 71984 , Cape Town, South Africa; 2 Clinical Operations Quality Management, IQVIA 59097 , Centurion, South Africa; 3 Strategic Health Innovation Partnerships (SHIP), South African Medical Research Council 59097 , Cape Town, South Africa; 4 Genetic Immunity Ltd., Budapest, Hungary; St. George's, University of London, London, United Kingdom

**Keywords:** nonlinear mixed-effects modeling, simulation, NONMEM, drug-susceptible TB

## Abstract

Ethambutol is used to treat tuberculosis (TB) in individuals living with HIV. Low concentrations of ethambutol have been reported in patients dosed with the World Health Organization (WHO)-recommended first-line regimen. We analyzed the pharmacokinetics of ethambutol in 61 HIV-positive individuals diagnosed with drug-sensitive TB enrolled in the tuberculosis and highly active antiretroviral therapy (TB-HAART) study. Participants started on TB treatment and were randomized to early or later introduction of efavirenz-based antiretroviral treatment. We explored potential covariate effects and evaluated the current WHO dosing recommendations for ethambutol in drug-susceptible and multidrug-resistant (MDR)-TB. A two-compartment model with first-order elimination allometrically scaled by fat-free mass and transit compartment absorption best described the pharmacokinetics of ethambutol. Clearance was estimated to be 40.3 L/h for a typical individual with a fat-free mass (FFM) of 42 kg. The Antib-4 formulation had 26% higher bioavailability and slower mean transit time by 37% compared with Rifafour. Simulations showed that individuals in the lower weight bands (<55 kg) who were administered ethambutol at WHO-recommended doses had relatively low drug exposures. These individuals would need doses of 825 mg if their body weight is <37.9 kg and 1,100 mg if it is between 38 and 54.9 kg to achieve the reference maximum concentrations of 2–6 mg/L and an area under the concentration-time curve (0–24) of 16–29 mg·h/L. To achieve these targets in MDR-TB treatment, a dose increment of 400 mg (extra tablet) would be required for individuals in the lower weight band (<46 kg). Our dose adjustments are consistent with the literature and can be recommended for consideration by the WHO for first-line drug-susceptible and MDR-TB treatment.

## INTRODUCTION

The World Health Organization (WHO) recommends for drug-susceptible tuberculosis (DS-TB), a two-phase, 6-month-long regimen that includes isoniazid and rifampicin for the entire treatment, with ethambutol and pyrazinamide taken during the first 2 months only (1). Ethambutol, as a first-line agent, is bacteriostatic and helps prevent resistance to other first-line anti-TB agents, such as rifampicin (2, 3). Ethambutol is normally administered as a hydrochloride (HCl) salt (4) at a recommended dose between 15 and 25 mg/kg (1). Ethambutol has an estimated oral bioavailability (*F*) of 80% and plasma protein binding of 70–80% (2, 3). About 50–70% of ethambutol is excreted unchanged in the urine (2, 3). After an ethambutol dose of 15–25 mg/kg, a peak concentration (Cmax) between 2 and 6 mg/L is reached after 2–4 h, and an area under the concentration curve from 0 to 24 h (AUC_0-24h_) of 16–29 mg·h/L (5, 6). This ethambutol exposure seems to be effective for a reported minimum inhibitory concentration (MIC) of 2 mg/L (1).

WHO has dosing recommendations for ethambutol targeting 15–25 mg/kg using a tablet strength of 275 mg for DS-TB (1) and 400 mg for MDR-TB (7), with both formulations containing the ethambutol HCl salt form. Despite the use of weight-band dosing, the WHO guidelines provide an inadequate dose adjustment for the effect of body size, as several authors report lower drug exposures in adults within the lower weight bands (8–10). These findings are in keeping with the well-established theory of allometry, which predicts a nonlinear relationship between clearance and body size (11), such that individuals with lower weight need a larger mg/kg dose to achieve the same AUC. Moreover, the descriptor of body size that more accurately correlates with drug clearance is often fat-free mass (FFM) (12), as opposed to total body weight (11, 13–15). FFM reflects body composition and can be estimated using total body weight, height, and sex (12). In a report by Muliaditan and Della Pasqua (14), individuals with body weight ≤40 kg, dosed according to WHO recommendations, achieved 20% lower AUC_0-24_ than participants in the higher weight bands. Similar findings have been observed with other anti-TB drugs, such as isoniazid (8, 9, 16, 17), pyrazinamide (8, 9, 16, 17), and rifampicin (8, 9, 16–18). The suboptimal exposure of ethambutol has been linked to high incidences of treatment failure, adverse drug reactions, and antibiotic resistance, particularly in patients living with HIV (8, 19). However, there are no reports on ethambutol interaction with antiretroviral therapy (ART) that could explain the suboptimal levels in patients with HIV. According to a systematic review conducted by Daskapan et al. (6), there is conflicting evidence on whether the presence of HIV causes pharmacokinetic (PK) variability of the first-line anti-TB drugs. Although some studies have shown no significant impact of HIV (20, 21), others have demonstrated a potential influence on *F* of anti-TB drugs (8, 22, 23).

We aimed to characterize the population PK of ethambutol in individuals newly diagnosed and treated for DS-TB and HIV, investigating available covariates with potential influence. Additionally, using Monte Carlo simulations of different dosing scenarios, we evaluated the achieved exposures across WHO weight bands.

## MATERIALS AND METHODS

### Pharmacokinetic data and study design

A detailed description of the tuberculosis-highly active antiretroviral therapy (TB-HAART) study population has been reported by McIlleron et al. (8). In brief, we enrolled newly diagnosed pulmonary DS- TB (sputum positive) ART-naive patients with HIV, aged 18–65 years old, between March 2007 and April 2008 at primary healthcare facilities in and around Durban, South Africa. All participants provided written informed consent. The University of Cape Town (UCT) Human Research Ethics Committee approved the study (HREC: 931/2023). TB and ART regimens were dosed according to weight-based bands as recommended in the WHO guidelines (1), consistent with the South African National TB treatment guidelines (24). Ethambutol was administered within the 4-in-1 fixed-dose combination formulation (FDC) containing 275 mg of ethambutol HCl, 75 mg of isoniazid, 400 mg of pyrazinamide, and 150 mg of rifampicin. Ethambutol doses were 550 mg for participants with a body weight of <37.9 kg, 825 mg for 38–54.9 kg, 1,100 mg for 55–70 kg, and 1,375 mg for weights >70 kg. Ethambutol formulations given during this study were e-275 Rifafour (Sanofi-aventis, South Africa) and Antib-4 (Rusan Pharma, India); both formulations had the same active ingredients, strength, and excipients according to the manufacturer’s brochures. Both ethambutol formulations contained 275 mg of ethambutol HCl (molecular weight of 277.23 g/mol), which corresponds to 202.68 mg of ethambutol (molecular weight of 204.31 g/mol) after adjusting by its salt fact of 0.737 (4). Participants were not switched between formulations throughout the study. The e-275 Rifafour was the primary formulation, and Antib-4 was used only when the primary formulation was not available (stock-out) at enrollment.

The study participants were enrolled and followed for 28 days, with PK visits on days 0, 7, 14, and 28 after TB treatment initiation. The participants were divided into 6 arms (stratified according to CD4+ T cell count and treatment, shown in (Table S1): arms 1 (220–349 CD4+ T cells/µL), 3 (350–500 CD4+ T cells/µL), and 5 (<200 CD4+ T cells/µL) included participants who were on TB treatment and started ART (efavirenz at 600 mg daily, lamivudine at 150 mg twice daily, and zidovudine at 300 mg twice daily) on day 13. Participants randomized to study arms 2 (220–349 CD4+ T cells/µL) and 4 (350–500 CD4+ T cells/µL) took only TB treatment for the entire duration of the study. Those in arm 6 only took ART and no anti-TB drugs; hence, they were not included in this analysis.

### Pharmacokinetic sampling and concentration analysis

The procedures of all study visits were identical. After an overnight fast, participants received the prescribed anti-TB drugs, including the assigned ethambutol formulation. Blood samples were drawn at 1, 1.5, 2, 4, 8, and 12 h post-dose. Predose samples were taken at 0 h on days 7, 14, and 28 and an additional sample on day 13 at the time of admission (i.e., at ~12 h before the day 14 PK assessment). ART was initiated on day 13 for participants allocated in arms that required anti-HIV treatment.

The analysis of the plasma samples was conducted in the facilities of UCT. The blood was spun down to separate the serum, which was stored at −80°C at the study site until transport to the UCT laboratory for the measurement of ethambutol concentrations. Ethambutol was assayed by liquid chromatography (LC)-tandem mass spectrometry on an API 4000 LC mass spectrometer using a modification of the method of Conte et al. (25). The mobile phase consisted of a gradient of 4 mM ammonium acetate in 0.05% trifluoroacetic acid and acetonitrile. Chromatography was performed on a Discovery HS F5 high-performance liquid chromatography (HPLC) column maintained at 25° C. Neostigmine served as the internal standard. Acetonitrile with 0.04% formic acid containing the internal standard was used to precipitate 50 µL of each sample, which was then centrifuged; 5 µL of the supernatant was injected into the column. The analytical lower limit of quantification (LLOQ) for ethambutol was 0.1 mg/L. Quality control analyses were performed both inter- and intra-day, with coefficients of variation (CV) remaining below 9% for all samples tested.

### Data analysis

Data were analyzed using nonlinear mixed-effects modeling software (NONMEM) (v. 7.5.1) with first-order conditional estimation with interaction (FOCE-I) (26, 27). Data handling and visualization were performed in R (v. 4.3.1), and diagnostics were generated using Xpose 4 and tidyvpc (v. 1.4.0). Visual predictive checks (VPCs) were generated using Pearl-speaks NONMEM (PsN, v 5.3.0) by performing 1000 simulations stratified according to study visits and statistically significant covariates for the final model after completing covariate modelling (26).

### Model development

Observed data were fitted into one- and two-compartment disposition models with first-order absorption and elimination. Different absorption delay approaches were tested, including lag time and transit absorption compartments. Between-subject variability (BSV) was evaluated for disposition parameters, and between-occasion variability (BOV) was evaluated for the absorption rate constant (Ka), *F*, and mean transit time (MTT). A combined error model (with additive and proportional components) was used to describe the residual unexplained variability (RUV). The concentrations below the LLOQ were imputed as half of the LLOQ (LLOQ/2) and their additive error was inflated by LLOQ/2 to reflect the larger uncertainty introduced by the imputation. The effect of body size was tested using allometric scaling by total body weight, fat, and FFM (12) on central and peripheral compartment parameters, with the exponents fixed to 0.75 and 1 for clearances and volumes, respectively. Additional participant baseline characteristics such as age, sex, weight, height, serum creatinine, creatinine clearance (CRCL) estimated using Cockcroft-Gault equation (28), CD4+ Tcell count, and time on treatment were included in the data and explored for covariate effects.

### Model evaluation

Statistical significance of model modifications was performed by comparing the NONMEM objective function value (OFV) using the likelihood ratio test at the 5% significance level (decrease in OFV ≥3.84 between nested models with one additional parameter). To evaluate the uncertainty of the parameter estimates, sampling importance resampling (SIR) was employed to obtain the 95% confidence intervals (CIs).

### Comparison of ethambutol models from the literature

In order to comprehensively compare published PK models for ethambutol, we conducted a thorough search of PubMed, Google Scholar, and ResearchGate databases to identify all relevant studies published to date. Our inclusion criteria encompassed published ethambutol models from studies involving adult populations over the age of 18, who were either healthy volunteers or being treated for TB, regardless of the type of TB and whether the patients were also diagnosed with HIV. Our review aimed to compare the clearance (CL) from all models related to the first-line ethambutol dose. First, all reported CLs were allometrically scaled for a 70 kg individual and divided by the reported value of *F* (if covariate effects were present in the analysis) to obtain apparent CL (CL/*F*). Finally, CL/*F* was adjusted for the ethambutol HCl salt factor of 0.737, if this adjustment was not mentioned in the original publication.

### Simulation

Using the PK parameter estimates from the final population PK model, we performed simulations in NONMEM to evaluate the exposures in different dosing scenarios and compare them with previously reported reference values. As summarized in (Table S2), the *in silico* population contained 1,225 virtual participants generated by repetition of characteristics from participants included in different studies on DS- or MDR-TB (6, 18, 27, 28), of whom 56.6% are men with a median (IQR) body weight of 52.1 (46.2–58.7) kg and FFM of 38.1 (34.3–42.4) kg, which were comparable with the current study population. A density plot for the weight distribution for the virtual/simulated participants is presented in (Fig. S1). Two different tablet sizes/doses were explored, the first-line FDC based on 275 mg ethambutol HCl tablet and the MDR-TB ethambutol HCl 400 mg tablet. WHO-recommended weight bands were used in the simulation for both DS-TB (25–37 kg, 38–54 kg, 55–70 kg, >70 kg) (1) and MDR-TB (30–35 kg, 36–45 kg, 46–55 kg, 56–70 kg, >70 kg) (7). Monte Carlo simulations aimed to compare the exposures to the reference values of 16–29 mg·h/L and 2–6 mg/L for AUC_0-24_ and Cmax, respectively (5, 6).

## RESULTS

### Pharmacokinetic data

PK data was available from 61 participants at the first visit, and their baseline characteristics are summarized in Table 1. Most participants were women, 33 (54.1%) with a median (IQR) weight of 55.2 (50.0–62.4) kg and FFM of 42 (36.3–45.5) kg. Most participants received an ethambutol dose of 825 mg (31 participants, 50.8%), 1,100 mg (19, 31.2%), 1,375 mg (10, 16.4%), and only 1 (1.6%) participant received 550 mg. Ethambutol doses remained unchanged throughout the study visits. The formulation e-275 Rifafour FDC was prescribed to 54 (88.5%) participants, and the remaining 7 participants (11.5%) took Antib-4. There were no significant differences in the baseline characteristics between arms. Not all participants completed the 4 visits. For days 7, 14, and 28, data were available from 58 (95%), 56 (92%), and 52 (85%) participants, respectively. The reasons for participation withdrawal were loss to follow-up (7 participants, 78%) and due to serious adverse events, including anemia and elevated amylase (2 participants, 22%). Among the 1,542 blood samples collected, 10 (0.65%) observations were below the limit of quantification (BLQ) and distributed in the pre-dose samples.

**TABLE 1:** Study participants’ demographics and characteristics*^c^*

Characteristics	Overall (*N* = 61)
Age (yr)	32.0 [27–37]
Female	33 (54.1)
Weight (kg)	55.2 [50–62.4]
Height (m)	1.59 [1.54–1.68]
Fat-free mass (kg)	42 [36.3–45.5]
CD4+ T cell count (cells/µL)	254 [159–323]
Creatinine clearance (mL/h)^*a*^	97 [80.1–117]
Type of formulation^*b*^	54 (88.5)
HIV positive	61 (100)
Started ART on day 13	41 (67.2)
Not on ART	20 (32.8)

^*a*^ Computed using the Cockroft-Gault equation (28).

^*b*^ Participants that took the primary formulation (e-275 Rifafour). ART, antiretroviral therapy.

^*c*^ Data are shown as median [interquartile range] or number of participants (percentage).

### Pharmacokinetic model

Ethambutol concentration-time data were best described by a two-compartment PK model with first-order absorption and three transit compartments (Fig. S2) . The two-compartment model proved to be significantly better than a one-compartment model (ΔOFV = 937, *P* < 0.001, 2 degrees of freedom [df]). The inclusion of absorption through transit compartments improved the model fit (ΔOFV = 193, *P* < 0.001) and was preferred to a lag time (ΔOFV = 43, *P* < 0.001). The estimation of the number of transit compartments (NN) was fixed to three transit compartments, which helped stabilize the parameter estimates of the model without affecting the model fit and no change in OFV was observed after fixing NN. A sensitivity analysis using fixed NN values of 3, 6, and 9 revealed comparable parameter estimates across models for different NN; however, the OFV was higher for models with NN values greater than 3. Allometric scaling, which was included at the initial stage of model building, improved the model fit. We found that a model with combined effect of FFM used on central parameters and total body weight on peripheral parameters performed better (ΔOFV = 23, *P* < 0.001) compared with models when weight (ΔOFV = 17, *P* < 0.001) or FFM (ΔOFV = 21, *P* < 0.001) was used alone in all the parameters. Additionally, the inclusion of FFM instead of total body weight as a descriptor of body size for CL explained a previously significant effect of female sex having lower CL. The pre-dose concentrations, which derive from self-reported doses, were characterized by larger variability compared with concentrations measured after observed doses administered at the clinic. We accounted for this by estimating a scaling factor increasing the BOV for all absorption parameters following unobserved doses taken before admission. This significantly improved the model fit (ΔOFV = 65, *P* < 0.001, 1 df) and was retained in the model.

**Fig 1:**
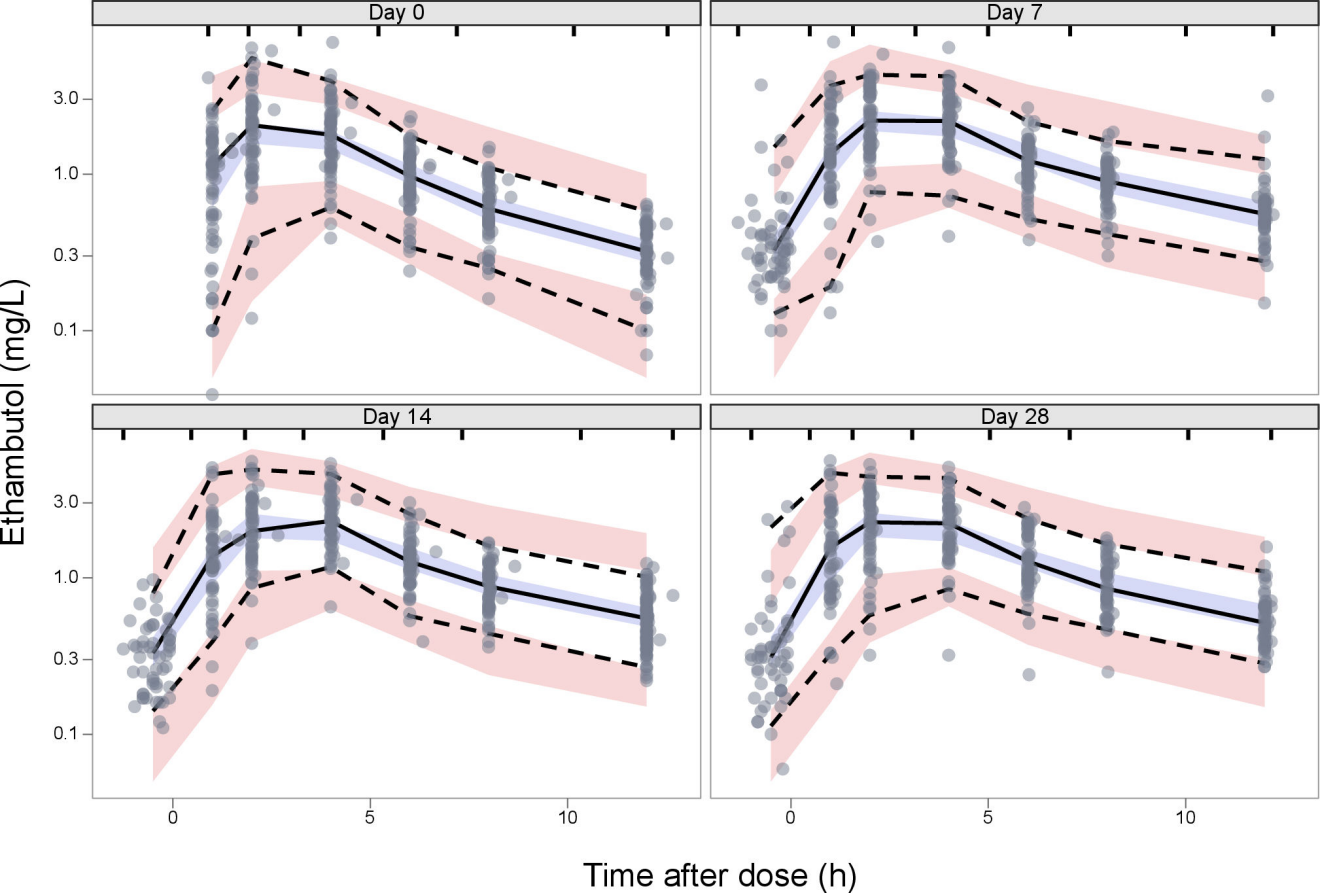
Visual predictive check stratified according to the four visits of the study (days 0, 7, 14, and 28). The gray circles represent the original data; dashed and solid lines are the 5th, 50th, and 95th percentiles of the original data, whereas the shaded areas are the corresponding 95% CIs for the same percentiles, as predicted by the model. The samples taken 12 h post-dose on day 13 were moved to +12 h post-dose (day 14) only for visualization of visual predictive checks.

The Antib-4 formulation was found to have 26% higher *F* (ΔOFV = 10.5, *P* < 0.01, 1 df) and reduced MTT by 37% (ΔOFV = 6.65, *P* < 0.01, 1 df) compared with Rifafour. Other tested covariates included CRCL, CD4+ T cell count, ART from day 13, and time on treatment, whose inclusion did not improve the model. We did not observe any significant trends when inspecting BOV on *F*, Ka, and MTT and BSV on CL over time. Additionally, we conducted tests to assess the variability between visits for parameters (CL, ka, MTT, and *F* ) and our findings showed that the differences between visits was not statistically significant. The goodness of fit of the final model was supported by diagnostic plots (Fig. S3). The VPCs of the final model showing a good data description by the model are presented in Fig. 1 and Fig. S4. The parameter estimates for the final model and the SIR 95% CIs are shown in Table 2. A summary of the comparison of our model CL and published models is presented in Table 3, showing that our value of CL is in line with the majority of previous studies.

**TABLE 2:** Covariate final model (parameter estimates and 95% CI)*^g^*

	Typical value (95%CI^*a*^)	Variability^*e*^ (95%CI^*a*^)
Clearance (L/h)^*c*^	34.8 (32.9–36.7)	BSV: 19.9 (16.7–23.5)
Central volume (L)^*c*^	156 (139–172)	
Absorption rate constant (1 /h)	0.877 (0.755–1.02)	BOV: 27.8 (25.2–30.3)
Bioavailability (F)	1 (FIXED)	BOV: 52.8 (45.4–61.3)
Mean transit time (h)	0.717 (0.641–0.788)	BOV: 66.3 (60.3–74.0)
Transit compartments (NN)	3 (FIXED)	
Intercompartmental clearance (L/h)^*d*^	34.4 (31.9–37.1)	
Peripheral volume (L)^*d*^	564 (489–663)	
Scaling factor for BOV of unobserved doses^*b*^ (-fold change)	2.20 (1.92–2.49)	
Antib-4 formulation effect on bioavailability (%)	+26.0 (12.0–41.3)	
Antib-4 formulation effect on mean transit time (%)	+37.1 (10.7–76.5)	
Proportional error (%)	24.8 (23.8–25.9)	
Additive error (mg/L)^*f*^	0.02 (FIXED)	

^*a*^ SIR was used to obtain the 95% CI.

^*b*^ This multiplicative factor applies to the BOV of the absorption parameters referring to doses not observed by the study staff, that is, taken at home on days before blood sampling. In the data set, the dose taken before the visit to the clinic and the predose concentration were defined as a separate PK occasion from the observed dose given in the clinic and the following concentrations.

^*c*^ Scaled to an individual with a fat-free mass (FFM) of 42 kg.

^*d*^ Scaled to an individual with a weight of 57 kg.

^*e*^ BSV, and BOV reported as approximate log-normal distribution (and calculated as $\text{CV}\%=\sqrt{\omega^{2}}\;\times 100$).

^*f*^ Fixed to 20% of the lower limit of quantification value (0.1 mg/L).

^*g*^ BSV, between-subject variability; BOV, between-occasion variability; RUV, residual unexplained variability.

**TABLE 3:** Comparison of previously published compartment models on ethambutol in adult population

Parameter	Study								
	Current study	Wijk et al. (29)	Peloquin et al. (30)	Jonsson et al. (31)	Denti et al. (15)	Mehta et al. (32)	Abdelwahab et al.^*a*^ (33)	Sekaggya-Wiltshire et al. (9)	Hall et al.^*b*^ (34)
Study participants	61	104	14	189	100	40	18	254	18
Study location	South Africa	South Africa	US	South Africa	Tanzania	Botswana	South Africa	Uganda	US
Study population	DS-TB and HIV	DS-TB	Healthy volunteers	DS-TB and HIV	DS-TB and HIV	DS-TB and HIV	DS-TB and HIV	DS-TB and HIV	Healthy volunteers
Dose adjusted for salt factor (0.737)	Adjusted	Adjusted	Not reported	Not reported	Not reported	Not reported	Not reported	Not reported	Not reported
Median weight (kg)	57	51.4	79.3	47	51.9	55	66	52	90.8
Clearance (L/h)	34.5	48.8	90	39.9	40.7	52.1	60.2	35.6	80.8
CL_std_ standardized to 70 kg (L/h)^*c*^	40.3	61.9	81.9	53.8	50.9	40.9	64.8	44.5	104.8
CL_std_ adjusted for salt factor (L/h)*^d^*	40.3	61.9	60.4	39.1	37.5	30.2	47.8	32.8	77.2

^*a*^ Study included only pregnant women as participants.

^*b*^ Study included only elderly participants.

^*c*^ CL_std_ = CL_orig_× (70 kg/ WT_orig_)^0.75^, where CL_orig_ and WT_orig_ are the value of CL and WT reported in the original study, whereas CL_std_ has been scaled to 70 kg.

^*d*^ Adjusted CL_std_ was obtained by multiplying CL_std_ by 0.737. DS-TB, drug-susceptible tuberculosis, HIV, human immunodeficiency virus. US, United States.

### Simulations

**Fig 2:**
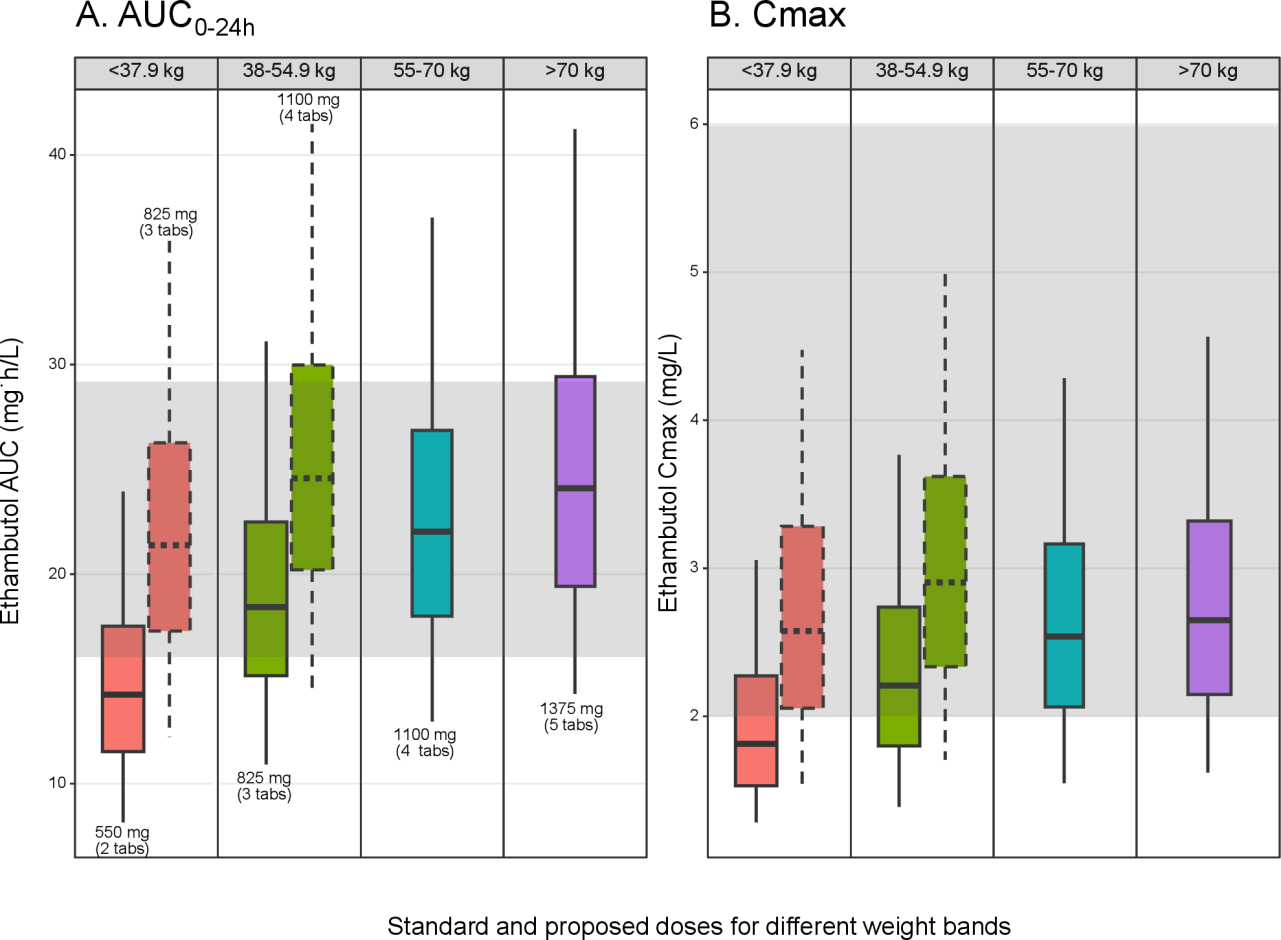
Comparison of simulated AUC_0–24_ and Cmax at steady state for standard and proposed first-line ethambutol dosing for a 275 mg tablet strength. Box plots depict the 25th, 50th, and 75th, the whiskers depict the 5th and 95th percentiles of the simulated data. The solid line boxplots represent exposure for standard WHO doses (mg, number of tablets), whereas the dashed line boxplots represent the proposed doses (mg, number of tablets). The gray-shaded horizontal area represents reference AUC_0–24_ (**A**) of 16–29 mg·h/L and Cmax (**B**) of 2–6 mg/L for the left and right panels, respectively. AUC = area under the concentration-time curve from time 0–24 h, Cmax = maximum concentration, mg = milligram, L = liter, h = hour.

The simulated ethambutol exposures obtained using the final PK model are shown in Fig. 2 and Fig. S5. Figure 2 shows the simulated ethambutol exposures achieved with the WHO weight-band-based regimen and the FDC formulation containing 275 mg of ethambutol. We found that simulated individuals with weights <55 kg exhibit an AUC_0-24_ comparable lower than those with weights ≥55 kg. Consequently, to balance the exposures with the reference AUC_0-24_ value and across the range of weights, we propose higher doses of ethambutol. The adjusted dose increases from 550 to 825 mg for weights ≤37.9 kg and for weights between 38 and 54.9 kg from 825 to 1,100 mg of ethambutol.

The same adjustments were made for the MDR-TB regimen using the 400 mg tablet (Fig. S5) with participants weighing <46 kg achieving lower median AUC_0-24_ compared with participants in higher weight bands. A dose increment from 800 mg to 1,200 mg was proposed. The dose recommendations are summarized in Table 4.

**TABLE 4:** Ethambutol dose recommendations*^a^*

Ethambutol tablet size/dose (mg)	Weight bands (kg)	WHO regimen (mg [number of tablets])	Proposed regimen (mg [number of tablets])
275 (formulation used for DS-TB)	<37.9	550 (2)	825 (3)
	38–54.9	825 (3)	1,100 (4)
	55–70	1,100 (4)	WHO regimen
	>70	1,375 (5)	
400 (formulation used for MDR-TB)	30–45.9	800 (2)	1,200 (3)
	46–70	1,200 (3)	WHO regimen
	>70	1,600 (4)	

^*a*^ DS-TB, drug-susceptible tuberculosis. MDR-TB, multidrug-resistant tuberculosis.

## DISCUSSION

We developed a population PK model of ethambutol in South African adults with DS-TB and HIV. The PK of ethambutol was best described using a two-compartment model with first-order absorption preceded by a series of transit compartments. The effect of body size was best captured by allometric scaling combining FFM on the central compartment and total body weight on the peripheral compartment. Simulations from our model show uneven exposure across weight bands when using the current WHO recommendations, with subjects of lower weight achieving lower exposures. We suggested an optimized strategy that increases doses in the lower weight bands balancing the exposures across weight range.

Our findings are not unexpected and consistent with a previous non-compartmental analysis of the same study cohort by McIlleron et al. (22), who reported observed ethambutol concentration to be reduced in low-weight and male subjects. They also showed a 1.4-fold AUC_0-12h_ increase in females weighing >75 kg compared with males weighing <35 kg. The decreased exposures in low body weight participants have been observed in other studies (8, 9, 14, 32) with all agreeing on weights <55 kg being the most affected. The explanation for this is that the relationship between body size and CL is known to be nonlinear (8, 11), meaning that weight-normalized CL is higher in smaller individuals, who will need higher doses to achieve the same exposure (9). As pointed out by Sekaggya-Wiltshire et al. (9), the WHO has acknowledged this effect in children and modified their recent guidance in children, recommending larger mg/kg doses for all first-line anti-TB drugs. However, the WHO recommendation of 15–25 mg/kg as the target dose for ethambutol remains unchanged in adults, along with similar recommendations for the other TB first-line agents.

In this analysis, we found that the best body size descriptor for ethambutol HCl is not total weight but FFM, which is consistent with the reports by both Abdelwahab et al. (33) and Wijk et al. (29) for ethambutol. FFM is estimated using weight, height, and sex (12, 33) and aims to adjust body weight for body composition to better approximate “PK body size.” FFM is closely related to metabolically active mass (35, 36) and thus correlates with the size of the organs involved in the elimination of drugs. In fact, the CL of drugs that are extensively metabolized by the liver is linked to liver volume (37, 38), and the higher number of hepatocytes, increased blood flow, and larger liver volume in males compared with females may account for the observed relationship between sex and drug CL (37). FFM is, on average, a smaller proportion of body weight in females, and, in fact, upon inclusion of FFM in the model, a previously observed sex effect, with females having lower CL than males, was no longer significant.

The fact that the best body size descriptor for CL is FFM further exacerbates the underexposure in smaller TB patients, as TB is often associated with weight loss. This means that TB patients in lower weight bands are often sicker and underweight (39, 40); hence, although their total body weight may be lower than that of patients in other weight bands, their FFM is generally not proportionally smaller. Therefore, the current WHO recommendations result in underexposures of arguably the most vulnerable patients, it has been demonstrated that patients with low body weight (<55 kg) have a 17–28% higher risk of death and TB treatment failure compared with those in higher weight bands (9). The WHO recommendations for ethambutol dosing MDR-TB also need attention as our simulations reported imbalances in exposure across weight bands, with the participants in the lower weight bands (<46 kg) again affected by underexposure (Fig. S5). It is important to note that although the WHO dosing guidelines are based on total body weight only and do not directly account for FFM or sex, we performed our simulations using an appropriate *in silico* population of TB patients that reflect their expected values of FFM in the smaller weight bands. We believe that with this strategy, we could still account for the most likely difference in body composition and size across the weight bands and obviate the limitations of dosing guidelines based on weight only.

Our results for ethambutol regarding the exposure imbalance across weight bands and the use of FFM for the scaling of CL are in line with previous findings on the other first-line TB drugs (rifampicin, isoniazid, and pyrazinamide) (9, 14, 16, 32). This means that the dosing adjustments we propose may be easily implementable for all first-line anti-TB agents using the current FDC tablets. It is in fact crucial to ensure that the concentrations of all the drugs in the FDC remain within the therapeutic range with the recommendation of adding one extra tablet. This has been evaluated in a previous report from 254 adult participants, which showed that increasing the dosage by one FDC tablet for participants weighing less than 55 kg did not lead to an increase in adverse events related to ethambutol, rifampicin, isoniazid, or pyrazinamide. In addition, all these drugs showed sufficient exposures (AUC and Cmax) across all weight bands (9). These findings were also supported by the study by McIlleron and Chirehwa, also adjusting for all first-line DS-TB drugs (16). Therefore, adapting the dosage as we propose is not expected to increase the risk of adverse events for the other first-line drugs contained in the 4-drug FDC.

Previous studies have evaluated the influence of HIV, CD4+ T cell count, efavirenz, HIV viral load, pregnancy, nutritional supplementation, age, sex, serum creatinine, or CRCL on ethambutol PK in adults. HIV (31), age (15), and study visits (pre- and post-antiretroviral therapy initiation, 33 days apart) (32) were found to be significant. The presence of HIV in TB patients is the most studied, with a considerable 27% reduction in the AUC_0-8h_ observed for ethambutol in participants with HIV, compared with participants without HIV (22). In addition, Jonsson et al. (31) reported HIV effect on *F*, with a 15.4% reduction in ethambutol’s *F*. There has been concern that patients with advanced HIV may have malabsorption due to HIV-induced enteropathy, hence the reduction in *F* of TB drugs (9). This was not possible to evaluate since all participants in our study were HIV positive, but reassuringly, we did not observe an effect of CD4+ cell count. We also did not observe an influence of ART on ethambutol PK starting at day 13.

If we compare our PK estimates with other models from the literature (after adjusting for the salt factor and standardizing to the same body weight using allometry), our value of CL for a typical 70 kg individual, 34.5 L/h, was similar (±20%) to previous reports in adults with TB (9, 15, 31–33). Interestingly, all three studies reporting CL values more than 50% higher than ours were conducted in either healthy volunteers or HIV-negative TB patients (29, 30, 34).

Our study highlighted that different FDC tablet formulations had an effect on ethambutol exposure; Antib-4 had 27.8% higher *F* compared to e-275 Rifafour, and also led to slower absorption. While it is reassuring that both formulations produced values of AUC_0-24h_ and Cmax values comparable to the reference ranges (AUC_0-24_ of 16–29 mg·h/L and Cmax of 2–6 mg/L), the 26% difference in *F* and 37% difference MTT is not expected between formulations that should be bioequivalent. In fact, the Antib-4 formulation was discontinued in South Africa due to quality concerns (41). This finding is concerning and not isolated for TB drugs. Others have also reported this issue in other first-line drugs like pyrazinamide (42) and rifampicin (22, 23, 42–44). For this reason, the need for bioequivalence studies should be emphasized, along with stringent monitoring of the quality of the FDC formulations manufacturing processes. Besides drug composition, several other factors as inappropriate packaging, particle size, and excipients (10, 45) can affect the absorption and decomposition of medications. All these considerations may have an influence on poor clinical outcomes by affecting plasma concentrations of the drugs (8, 9, 32).

Our study has some limitations. Assessment of the impact of CRCL in the PK of ethambutol was not possible, likely to be related to the stable renal function in all participants in the study. Our study did not evaluate safety endpoints such as optic neuritis that may be associated with increasing ethambutol exposures as it was not designed and powered for that purpose. Another limitation is that body weight was only recorded at baseline. While significant increases in body weight are expected in the first weeks of treatment, we believe that the balanced design of the study (whereby all patients were sampled at 4 scheduled visits from treatment initiation to 4 weeks with minimum dropout) mitigates the consequences of this lack of information. Finally, all the TB patients in our study were HIV positive. Reassuringly we could not detect any effect of ART or CD4+ T cell count. On the other hand, a strength of the study was that it had a relatively large sample size, and intensively sampled PK profiles were available from 4 separate visits.

### Conclusion

In summary, we conducted a study on the population PK of ethambutol in South African TB patients living with HIV and found that the currently recommended weight-based regimens by WHO for first-line ethambutol dose led to inadequate drug exposure for lower body weight patients (<55 kg), with high variability across weight bands. For DS-TB patients with a weight below 55 kg who were prescribed a first-line ethambutol dose of 275 mg, it is recommended to increase their dosage by one FDC tablet for optimal and better-balanced exposures compared with heavier patients. Similarly, patients with MDR-TB who weigh <46 kg and are given a 400 mg ethambutol tablet should also increase their dosage by one additional 400 mg tablet. These proposed dose increments for ethambutol are similar to those previously recommended for the companion drugs in the first-line treatment, and the WHO should consider adjusting dose recommendations, especially for lower weight bands. Our findings also suggest that the use of different anti-TB FDC formulations affected the exposure and absorption of ethambutol. This highlights the need for pharmaceutical companies and regulatory authorities to ensure thorough bioequivalence studies.

## SUPPLEMENTAL MATERIAL

Supplemental material aac.01200-24-s0001.docx Tables S1 and S2; Fig. S1 to S5.

ASM does not own the copyrights to Supplemental Material that may be linked to, or accessed through, an article. The authors have granted ASM a non-exclusive, world-wide license to publish the Supplemental Material files. Please contact the corresponding author directly for reuse.
