# Supplementary material for: Pharmacokinetics of ethambutol and weight banded dosing in South African adults newly diagnosed with tuberculosis and HIV
Source: Antimicrob Agents Chemother. 2024 Dec 23;69(2):e01200-24. doi: 10.1128/aac.01200-24 (PMC11823665; doi:10.1128/aac.01200-24)
Supplement: Supplemental material — Tables S1 and S2; Fig. S1 to S5. [file aac.01200-24-s0001.docx]

**Pharmacokinetics of ethambutol and weight banded dosing in South African adults newly diagnosed with tuberculosis and HIV.**

**Supplementary information.**

**Tables with legends.**

Table S1. Summary of study arms based on treatment and CD4+ T cell count.

| CD4+ T-cell count (cells/μL) | Treatment | | |
| --- | --- | --- | --- |
|  | Anti-TB + ART | Anti-TB | ART |
| 350-500 | (1) | (2) |  |
| 220-349 | (3) | (4) |  |
| < 200 | (5) |  | (6) |

TB, tuberculosis, ART, antiretroviral therapy. Numbers (1-6) indicates the study arms (group of participants) receiving the type of treatment from the CD4+ T cell count category. Arm 6 (CD4+ T cell count <200 cells/μL ) with participants receiving only ART were excluded from the study.

Table S2. Demographic data for virtual simulation population.

| Characteristics | Overall (N=1225) |
| --- | --- |
| Males | 690 (56.3) |
| Weight (kg) | 52.4 [46.2-58.7] |
| Height (m) | 1.64 [1.58-1.70] |
| Fat-free mass (kg) | 38.1 [34.3-42.4] |

Data shown as the median and interquartile range (IQR) or number of participants and percentage.

**Figures with legends.**

**
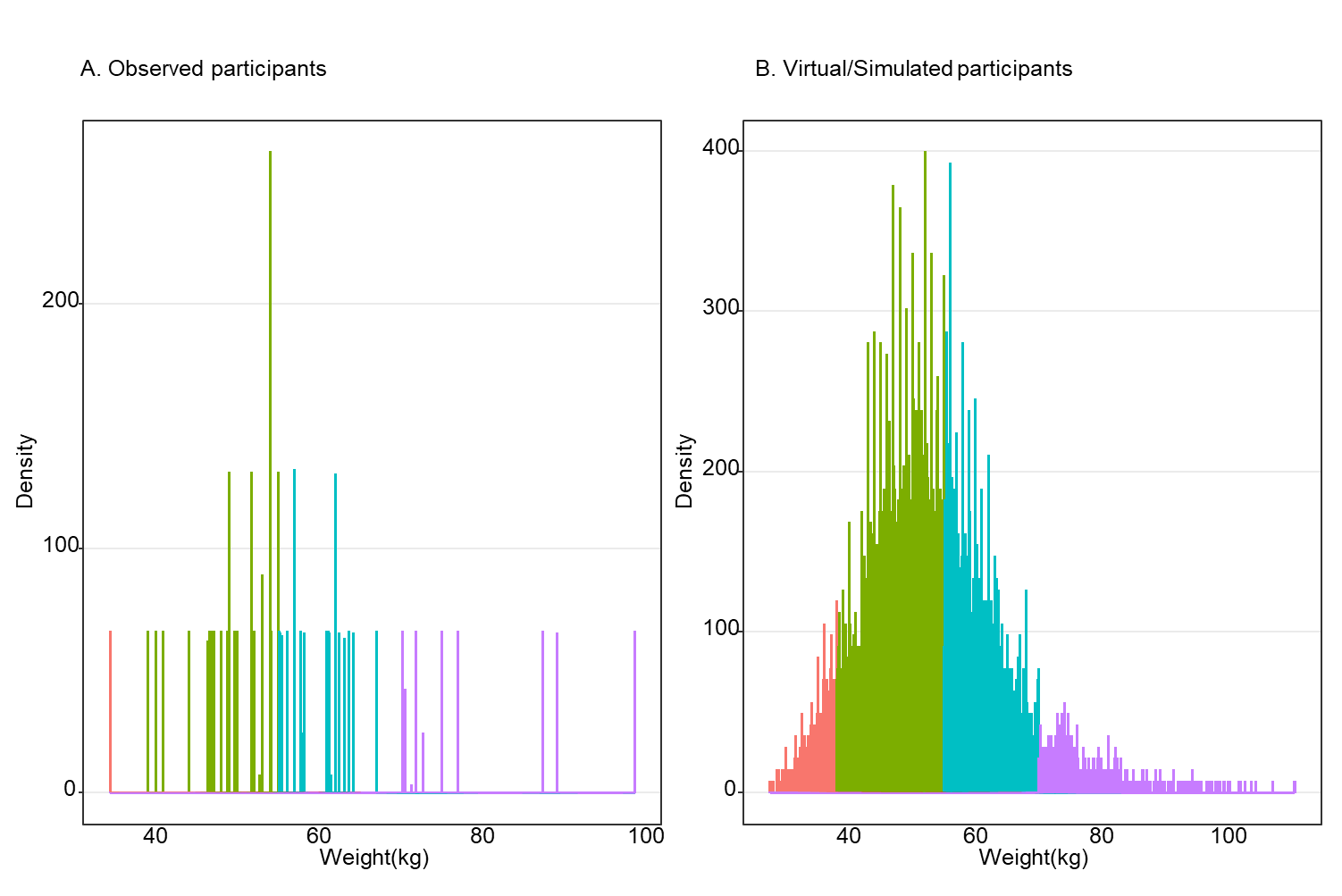
**

Figure S1. Density plot for weight distribution in virtual participants (right panel) and the study participants (left panel).


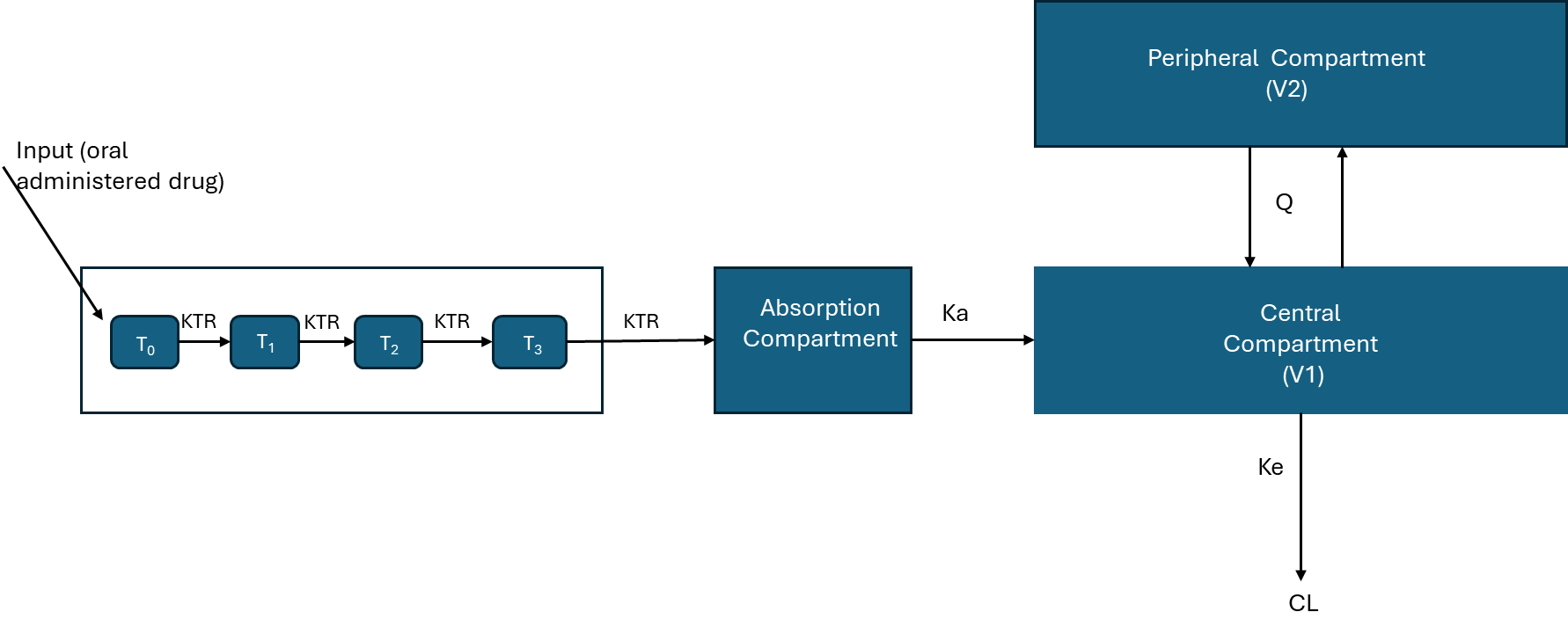
 Figure S2. A schematic representation of the selected PK ethambutol model. The dose of ethambutol is assumed to go through a series of transit compartments (T_0-3_) before reaching the absorption site, from which it is absorbed into the central compartment and distributed to the peripheral compartment. It is then eliminated from the central compartment with first-order kinetics. KTR = transit rate constant, Ka = absorption rate constant, V1= central volume, V2 = peripheral volume, Q = intercompartmental clearance, CL= clearance.


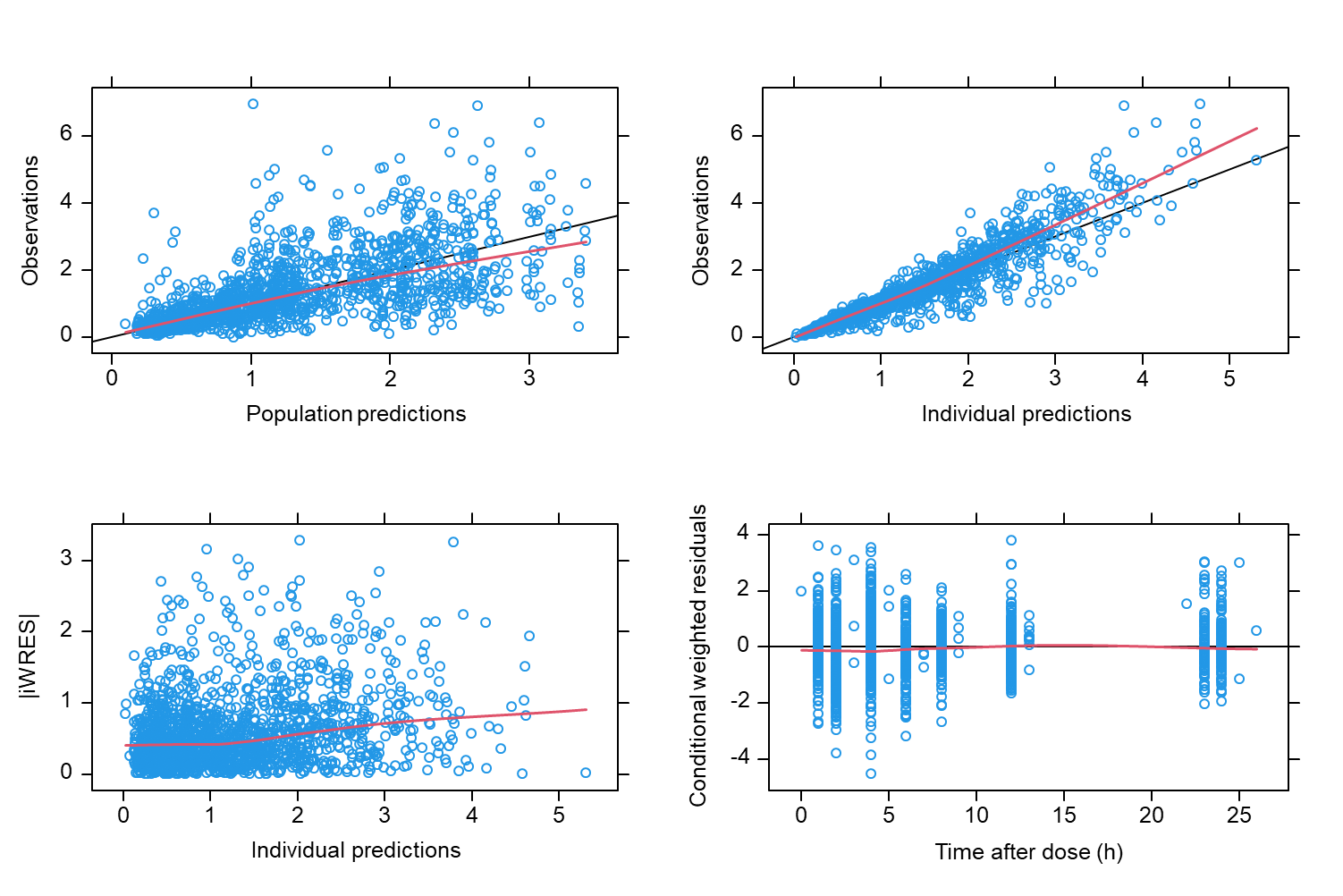


Figure S3. Goodness-of-fit plots for the final model. Key diagnostic plots for all four visits and overall plots. In each plot, symbols are data points, the solid red line is a line with slope 1 or 0 and the solid red line is a Loess smoothed line. IWRES, individual weighted residuals.

1. Stratified according to formulation.


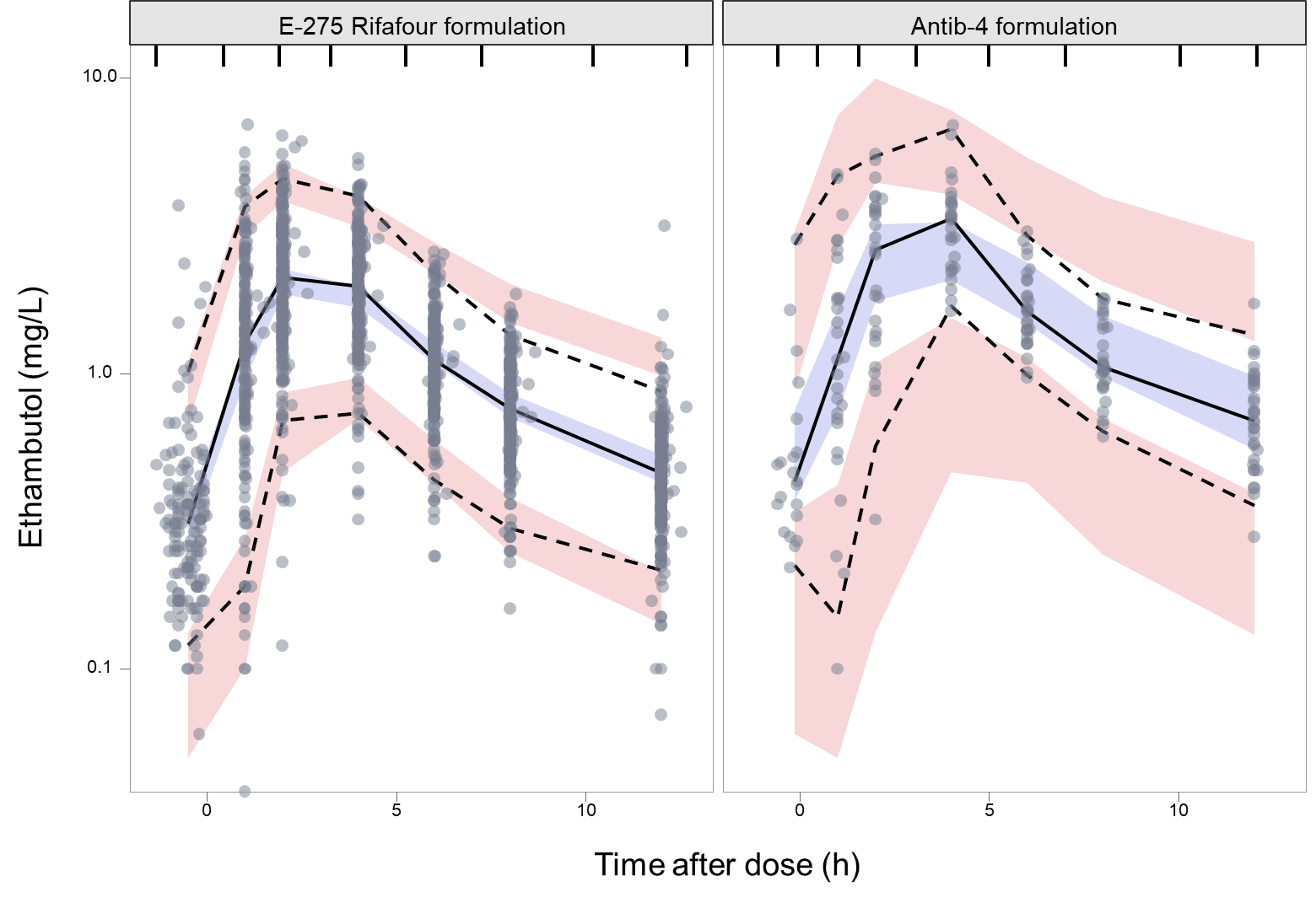


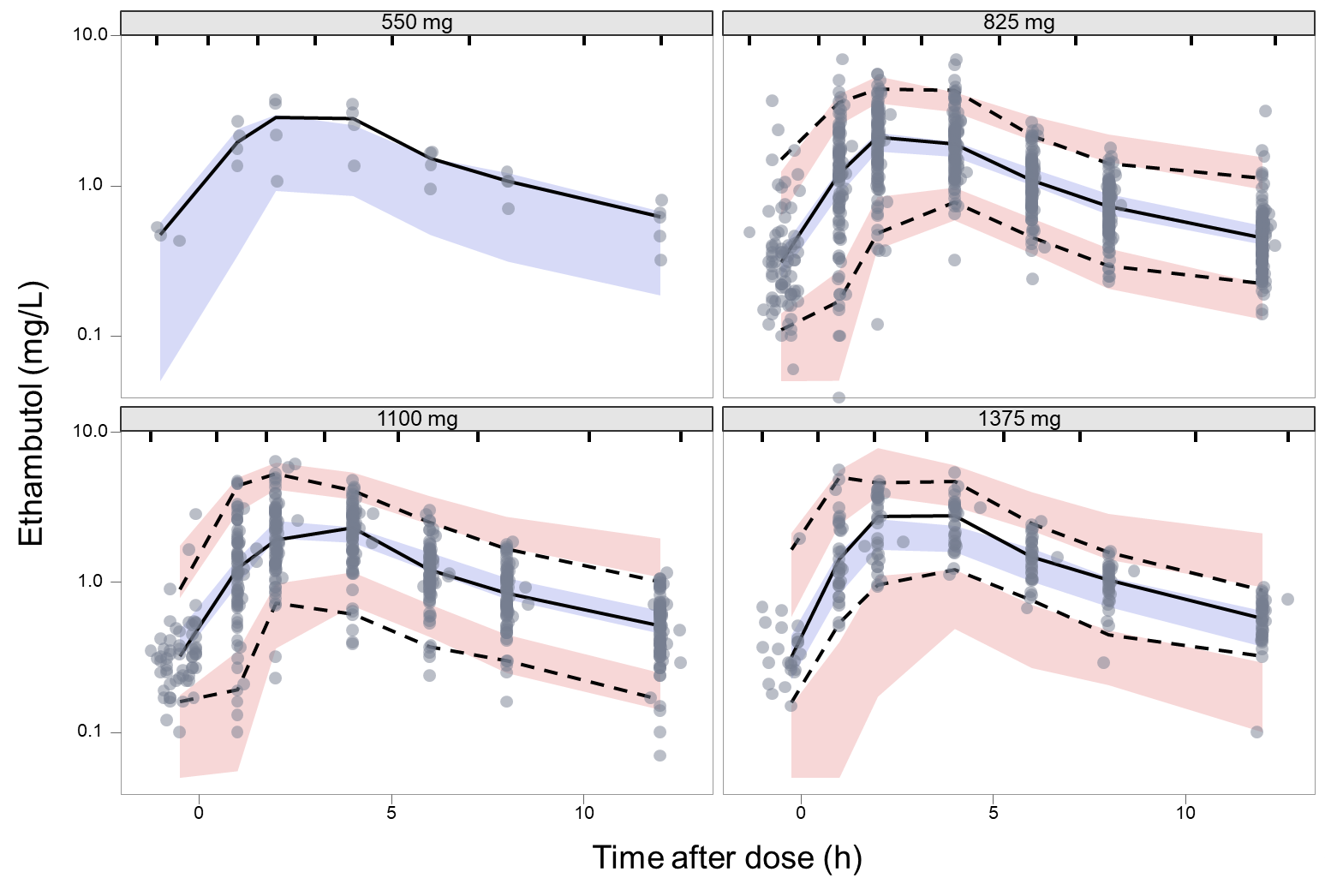


1. Stratified according to dose.

Figure S4. Visual predictive check stratified according to the formulation (A) and dose (B). The grey circles represent the original data, dashed and solid lines are the 5^th^, 50^th^, and 95^th^ percentiles of the original data, while the shaded areas are the corresponding 95% confidence intervals for the same percentiles, as predicted by the model. The 550 mg panel had less participants, and only median (50^th^ percentile) was shown without the 5^th^ and 95^th^ percentiles. The samples taken at -12h (pre-day samples) at day 13, were moved to +12 h (day 14 PK assessment) for only the purpose of these plots (VPC). The pre-day samples were only done in day 13, so movement of this concentrations only affects day 14 samples.


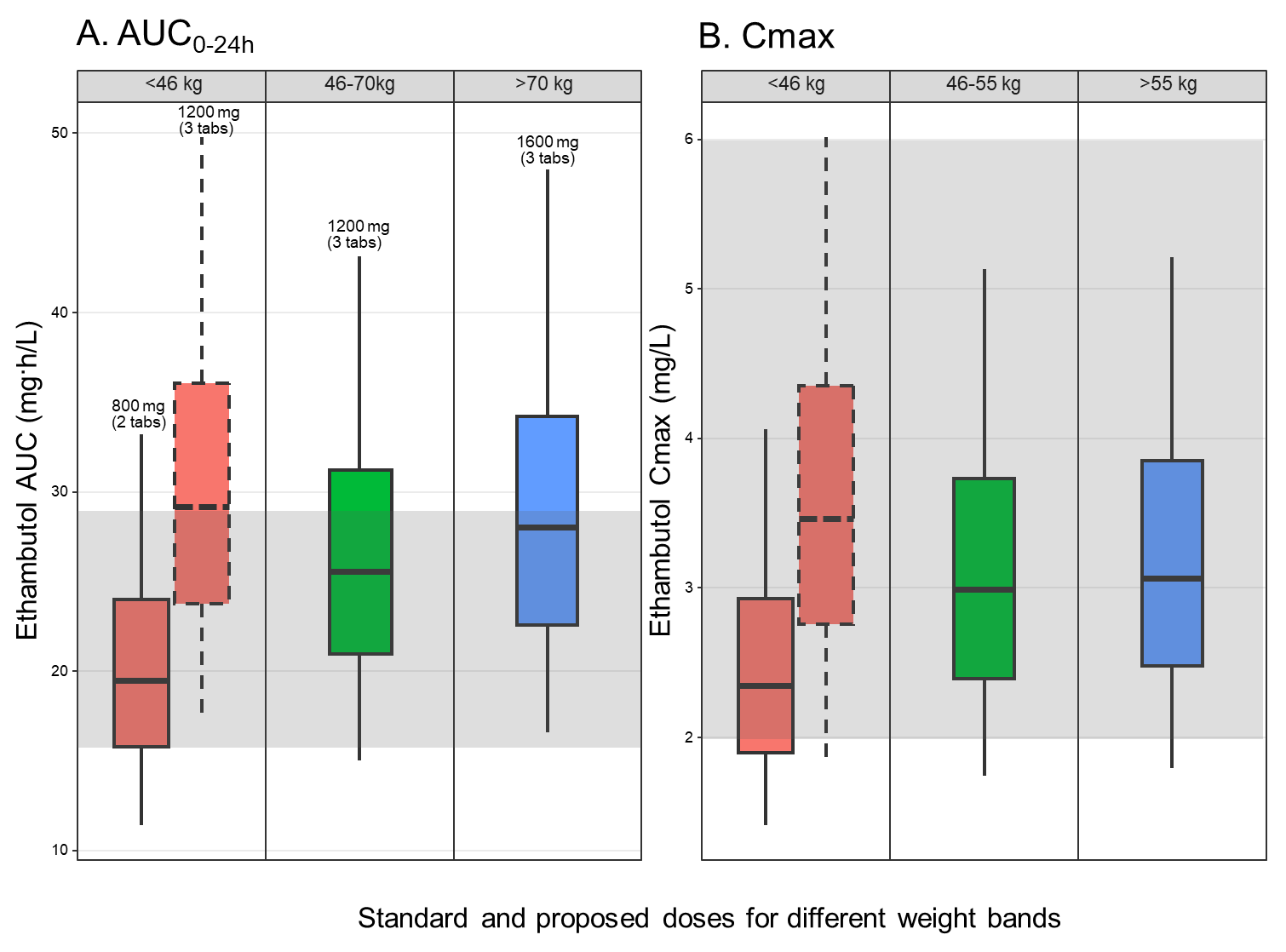


Figure S5. Comparison of simulated AUC_0–24_ and Cmax at steady-state for standard and proposed MDR-TB ethambutol dosing for a 400 mg tablet strength. Box and whisker plots depict the 5th, 25th, median, 75th, and 95th percentiles of the simulated data. The solid line boxplots represent exposure for standard WHO doses (mg (number of tablets)), while the dashed line boxplots represent the proposed doses (mg (number of tablets)). The shaded horizontal area represents reference AUC_0–24_ (A) of 16-29 mg·h/L and Cmax (B) of 2-6 mg/L for the top and bottom panels, respectively. AUC= area under the concentration-time curve from time 0–24 h, Cmax=maximum concentration, mg=milligram, L=litre, h=hour

**NONMEM model control file for ethambutol**

$PROBLEM Ethambutol

$INPUT ID TIME OCC VISIT VPCTIME AMT MDV EVID CENS BLQ DV ARM

SEX_F1 HEIGHT WEIGHT FORM SCCREAT CD4COUNT HAART OBSERVED

AGE CRCL EFZ

$DATA Ethambutol_20230823.csv IGNORE=@

$ABBREVIATED COMRES=2 ;

$SUBROUTINE ADVAN5 TRANS1 ;TOL=9 ;SSTOL=5 SSATOL=5 ATOL=9

$MODEL NCOMPARTMENTS=7 ; 4

COMP=(TRANSIT1,DEFDOSE) ; 1 GUT TRANSIT 1

COMP=(TRANSIT2) ; 2 GUT TRANSIT 2

COMP=(TRANSIT3) ; 3 GUT TRANSIT 3

COMP=(TRANSIT4) ; 4 GUT TRANSIT 4

COMP=(ABS) ; 5 GUT ABSORPTOIN

COMP=("CENTRAL",DEFOBSERVATION) ; 6 CENTRAL

COMP=(PERI1) ; 7 PERIPHERAL

$PK

; ------- BSV------------------------------------------------------------------------------------------------------

BSVCL = ETA(1)

BSVV = ETA(2)

BSVKA = ETA(3)

BSVBIO = ETA(4)

BSVV3 = ETA(5)

BSVQ = ETA(6)

BSVV4 = ETA(7)

BSVQ2 = ETA(8)

BSVMTT = ETA(9)

BOVCL = 0

BOVBIO = 0

BOVKA = 0

BOVMTT = 0

IF (OCC==1) THEN

BOVCL = ETA(10)

BOVKA = ETA(17)

BOVBIO = ETA(24)

BOVMTT = ETA(31)

ENDIF

IF(OCC==2) THEN

BOVCL = ETA(11)

BOVKA = ETA(18)

BOVBIO = ETA(25)

BOVMTT = ETA(32)

ENDIF

IF (OCC==3) THEN

BOVCL = ETA(12)

BOVKA = ETA(19)

BOVBIO = ETA(26)

BOVMTT = ETA(33)

ENDIF

IF(OCC==4) THEN

BOVCL = ETA(13)

BOVKA = ETA(20)

BOVBIO = ETA(27)

BOVMTT = ETA(34)

ENDIF

IF (OCC==5) THEN

BOVCL = ETA(14)

BOVKA = ETA(21)

BOVBIO = ETA(28)

BOVMTT = ETA(35)

ENDIF

IF(OCC==6) THEN

BOVCL = ETA(15)

BOVKA = ETA(22)

BOVBIO = ETA(29)

BOVMTT = ETA(36)

ENDIF

IF (OCC==7) THEN

BOVCL = ETA(16)

BOVKA = ETA(23)

BOVBIO = ETA(30)

BOVMTT = ETA(37)

ENDIF

;---------ExtraBOV for observed data

ExtraBOV = THETA(13)

IF (OBSERVED==0) THEN

BOVCL = BOVCL*ExtraBOV

BOVKA = BOVKA*ExtraBOV

BOVBIO = BOVBIO*ExtraBOV

BOVMTT = BOVMTT*ExtraBOV

ENDIF

;---------Between visit effect for KA/BIO etc---------------------------------------

BVVCL = 0

IF (VISIT == 1) BVVCL = ETA(38)

IF (VISIT == 2) BVVCL = ETA(39)

IF (VISIT == 3) BVVCL = ETA(40)

IF (VISIT == 4) BVVCL = ETA(41)

; -------------- Calculation of Fat-free Mass

; These formulas require WT in KG and HT in m !!!

; Conversion from cm to m

HTM = HEIGHT

WT = WEIGHT

IF (SEX_F1.EQ.1) THEN ; female

WHSMAX=37.99

WHS50=35.98

ELSE ;males

WHSMAX=42.92

WHS50=30.93

ENDIF

HTM2 = HTM**2

FFM = (WHSMAX*HTM2*WT)/(WHS50*HTM2+WT)

FAT = WT-FFM

IF (FAT.LT.0) FAT = 0

; ------- Typical values of covariates

TVWT = 57

TVFAT = 15

TVFFM = 42

;--------- Allometric scaling and covariates

ALLMCL_WT = (WT/TVWT)**0.75

ALLMV_WT = (WT/TVWT)

ALLMCL_FAT = (FAT/TVFAT)**0.75

ALLMV_FAT = (FAT/TVFAT)

ALLMCL_FFM = (FFM/TVFFM)**0.75

ALLMV_FFM = (FFM/TVFFM)

;----------------------------------Formulation on BIO---------------------------------------

IF(FORM.EQ.1) TVBIO = THETA(4)

IF(FORM.EQ.2) TVBIO = THETA(4)*(1 + THETA(14))

;-------------------Formulation on MTT____________________-------------------------------

IF(FORM.EQ.1) TVMTT = THETA(7)

IF(FORM.EQ.2) TVMTT = THETA(7)*(1 + THETA(15))

;---------Typical values-----------------------------------------------------------------------------------------

TVCL = THETA(1)*ALLMCL_FFM

TVV = THETA(2)*ALLMV_FFM

TVKA = THETA(3)

;TVBIO = THETA(4)

;TVMTT = THETA(7)

TVV3 = THETA(8)*ALLMV_WT

TVQ = THETA(9)*ALLMCL_WT

TVV4 = THETA(10);*ALLMV_WT

TVQ2 = THETA(11);*ALLMCL_WT

TVNN = THETA(12)

;-----------Define parameters-----------------------------------------------------------------------------------

CL = TVCL*EXP(BSVCL+BOVCL+BVVCL) ; CLEARANCE

V = TVV*EXP(BSVV) ; CENTRAL VOL.

KA = TVKA*EXP(BSVKA+BOVKA) ; ABS. RATE CONSTANT

BIO = TVBIO*EXP(BSVBIO+BOVBIO) ; BIOAVAILABILITY

MTT =TVMTT*EXP(BSVMTT+BOVMTT) ; MTT TIME

V3 = TVV3*EXP(BSVV3) ; PERIPH VOL

Q = TVQ*EXP(BSVQ) ; INTER COMPT CL

V4 = TVV4*EXP(BSVV4) ; PERIPH VOL2

Q2 = TVQ2*EXP(BSVQ2) ; INTER COMPT CL2

NN = TVNN ; Number of transit compartments

;-----------------------------------------------------------------------------------------------------------

; re-parameterization

F1 = BIO

KTR = (NN+1)/MTT ; Calculation of KTR

K12 = KTR ; TRANSIT 1

K23 = KTR ; TRANSIT 2

K34 = KTR ; TRANSIT 3

K45 = KTR ; TRANSIT 4

K56 = KA ; Rate of ABSORPTION

K60 = CL/V ; rate constant of elimination)

K67 = Q/V ; rate constant from central to peripheral 1

K76 = Q/V3 ; rate constant from peripheral 1 to central

S6 = V ; Scalling based on central compartment

;;;--------------------------------------------------------------

A_0(1) = 1E-12

A_0(2) = 1E-12

A_0(3) = 1E-12

A_0(4) = 1E-12

A_0(5) = 1E-12

A_0(6) = 1E-12

A_0(7) = 1E-12

;================================================================;

$ERROR

IPRED=A(6)/V

LLOQ = 0.1

CENS_THR = LLOQ

PROP = IPRED*THETA(5)

ADD = THETA(6)+(CENS_THR*0.2)

IF (ICALL/=4.AND.CENS==1) THEN

ADD = ADD +(CENS_THR*0.5)

ENDIF

NO_FIT = 0

IF (ICALL/=4.AND.CENS==2) THEN

PROP = 0

ADD = 10000000000

NO_FIT = 1

ENDIF

W = SQRT(ADD**2+PROP**2)

IF (W.LE.0.000001) W=0.000001

IRES=DV-IPRED

IWRES=IRES/W

Y = IPRED + W*ERR(1)

IF (ICALL==4.AND.Y<=CENS_THR) Y = CENS_THR/2

; To calculate time after dose.

IF(AMT>0) THEN

TIMEDOSE = TIME

AMOUNTDOSE = AMT

ENDIF

TAD = TIME-TIMEDOSE

VPCTIME2= TIME - TIMEDOSE

IF(VPCTIME2.GE.50) THEN

VPCTIME2 = VPCTIME2 - 72

ENDIF

VARCL = BSVCL + BOVCL

VARBIO = BSVBIO + BOVBIO

VARAUC = BSVBIO + BOVBIO - BSVCL - BOVCL

VARABS = BOVKA + BSVKA -BSVMTT - BOVMTT ;- BSVLAG - BOVLAG ;

;------------------------------------------RETRIEVE AMOUNT IN EACH COMPARTMENT----------

AA1 = A(1)

AA2 = A(2)

AA3 = A(3)

AA4 = A(4)

AA5 = A(5)

AA6 = A(6)

AA7 = A(7)

;--------------------------------------------------------------------------------------------------------------------

$THETA (0,34.2408,90) ; 1 CL [L/h]

$THETA (0,154.296,800) ; 2 V [L]

$THETA (0,0.869334,5) ; 3 KA [1/h]

$THETA 1 FIX ; 4 BIO

$THETA (0,0.25289,0.5) ; 5 PROP []

$THETA 4.37464E-007 FIX ; 6 ADD [mg/L]

$THETA (0,0.734944,3) ; 7 MTT

$THETA (0,598.167,800) ; 8 V3 [L]

$THETA (0,35.228,200) ; 9 Q [L/h]

$THETA (0,0,800) FIX ; 10 V4 [L]

$THETA (0,0,90) FIX ; 11 Q2 [L/h]

$THETA 3 FIX ; 12 NN []

$THETA (0,2.16367,10) ; 13 ExtraBOV []

$THETA (-1,0.268473,3) ; 14 FORM_BIO

$THETA (-1,0.01,3) ; 15 FORM_MTT

;--------------------------------------------------------------------------------------------------------------------

$OMEGA BLOCK(1) 0.0391723 ; 1 BSV CL

$OMEGA BLOCK(1) 0 FIX ; 2 BSV V

$OMEGA BLOCK(1) 0 FIX ; 3 BSV KA

$OMEGA BLOCK(1) 0 FIX ; 4 BSV BIO

$OMEGA BLOCK(1) 0 FIX ; 5 BSVV3

$OMEGA BLOCK(1) 0 FIX ; 6 BSVQ

$OMEGA BLOCK(1) 0 FIX ; 7 BSVV4

$OMEGA BLOCK(1) 0 FIX ; 8 BSVQ2

$OMEGA BLOCK(1) 0 FIX ; 9 BSVMTT

;---------------------------------------------------------------------------------------------------------------------------------------------------------------------

$OMEGA BLOCK(1) 0 FIX ; 0 BOVCL

$OMEGA BLOCK(1) SAME

$OMEGA BLOCK(1) SAME

$OMEGA BLOCK(1) SAME

$OMEGA BLOCK(1) SAME

$OMEGA BLOCK(1) SAME

$OMEGA BLOCK(1) SAME

;----------------------------------------------------------------------------------------------------------------------------------------------------------------------

$OMEGA BLOCK(1) 0.26492 ; 17 BOVBIO

$OMEGA BLOCK(1) SAME

$OMEGA BLOCK(1) SAME

$OMEGA BLOCK(1) SAME

$OMEGA BLOCK(1) SAME

$OMEGA BLOCK(1) SAME

$OMEGA BLOCK(1) SAME

;---------------------------------------------------------------------------------------------------------------------------------------------------------------------

$OMEGA BLOCK(1) 0.0729173 ; 24 BOVKA

$OMEGA BLOCK(1) SAME

$OMEGA BLOCK(1) SAME

$OMEGA BLOCK(1) SAME

$OMEGA BLOCK(1) SAME

$OMEGA BLOCK(1) SAME

$OMEGA BLOCK(1) SAME

;---------------------------------------------------------------------------------------------------------------------------------------------------------------------

$OMEGA BLOCK(1) 0.494096 ; 31 BOVMTT

$OMEGA BLOCK(1) SAME

$OMEGA BLOCK(1) SAME

$OMEGA BLOCK(1) SAME

$OMEGA BLOCK(1) SAME

$OMEGA BLOCK(1) SAME

$OMEGA BLOCK(1) SAME

;---------------------------------------------------------------------------------------------------------------------------------------------------------------------

$OMEGA BLOCK(1) 0 FIX ; 38 BVVCL

$OMEGA BLOCK(1) SAME

$OMEGA BLOCK(1) SAME

$OMEGA BLOCK(1) SAME

;--------------------------------------------------------------------------------------------------------------------------------------------------------

$SIGMA 1 FIX

;-------------------------------------------------------------------------------------------------------------------------------------------------------

$ESTIMATION MSFO=run802b.msf METHOD=1 INTER MAXEVAL=9999 PRINT=1 NOABORT ;MCETA=500 RANMETHOD=4P SADDLE_RESET=1 REPEAT

NSIG=3 SIGL=9

NONINFETA=1 ETASTYPE=1 PRINT=1

$COVARIANCE PRINT=E

;--------------------------------------------------------------------------------------------------------------------

$TABLE ID OCC TIME MDV TAD VISIT AA1 AA2 ; AA3 AA4

Y DV PRED RES WRES IPRED IRES IWRES CWRES OBJI WRESCHOL

NOPRINT NOAPPEND ONEHEADER FORMAT=, FILE=sdtab802b.csv

;--------------------------------------------------------------------------------------------------------------------

$TABLE ID OCC TIME MDV CL V KA BIO MTT NN V3 Q V4 Q2 BSVMTT BSVCL

BSVV BSVKA BSVBIO BSVV3 BSVQ BSVV4 BSVQ2 BOVMTT BOVCL

BOVKA BOVBIO VARCL VARBIO VARAUC VPCTIME2 BVVCL NOPRINT

NOAPPEND ONEHEADER FORMAT=, FILE=patab802b.csv

;--------------------------------------------------------------------------------------------------------------------

$TABLE ID OCC SCCREAT CD4COUNT WEIGHT HEIGHT FFM FAT NOPRINT

NOAPPEND ONEHEADER FORMAT=, FILE=cotab802b.csv

;--------------------------------------------------------------------------------------------------------------------

$TABLE ID OCC SEX_F1 HAART VISIT FORM ARM EFZ NOPRINT NOAPPEND

ONEHEADER FORMAT=, FILE=catab802b.csv

;--------------------------------------------------------------------------------------------------------------------

$TABLE ID OCC TIME MDV TAD VISIT AA1 AA2 ; AA3 AA4

Y DV PRED RES WRES IPRED IRES IWRES CWRES OBJI CL V KA BIO

MTT NN V3 Q V4 Q2 BSVCL BSVV BSVKA BSVBIO BSVV3 BSVQ BSVV4

BSVQ2 BOVCL BOVKA BOVBIO BOVMTT BSVMTT VARCL VARBIO VARAUC

WEIGHT HEIGHT FFM FAT SEX_F1 VPCTIME2 BVVCL NOPRINT

NOAPPEND ONEHEADER FORMAT=, FILE=mytab802b.csv

;--------------------------------------------------------------------------------------------------------------------
